# Exploring Entrepreneurial Behavior and Model Innovation of New Ventures *via* News Communication

**DOI:** 10.3389/fpsyg.2021.730299

**Published:** 2021-11-23

**Authors:** Ningfeng Sun, Gang Zhu, Hanning Song, Fengrui Zhang, Yuanbing Liu

**Affiliations:** ^1^School of Humanities, Southwestern University of Finance and Economics, Chengdu, China; ^2^Chinese Academy of International Trade and Economic Cooperation, Beijing, China; ^3^School of Banking and Finance, University of International Business and Economics, Beijing, China; ^4^College of Life Sciences, Sichuan Agricultural University, Yaan, China; ^5^College of Teacher, Jiaxing University, Jiaxing, China

**Keywords:** new ventures, entrepreneurial behavior, entrepreneurial self-efficacy, news communication, model innovation

## Abstract

The investigation into entrepreneurial behavior and model innovation of new ventures based on news communication aims to reinforce the market competition strength and improve the performance level of new ventures to meet the urgent needs of transformation and upgrading. Based on the theoretical basis of news communication and social cognition theory, a theoretical model is constructed to analyze the relationship between entrepreneurial behavior, innovation mode, and entrepreneurial self-efficacy (ESE) and to provide a reference for the implementation of entrepreneurial behavior of new ventures. Evidently, taking individual factors as antecedent variables to verify their impact on internal entrepreneurial behavior extends the scope of research on corporate entrepreneurship and also provides significant managerial implications for the promotion of entrepreneurial activities.

## Introduction

Under the background of the new normal economy, Premier Li Keqiang puts forward the concept of “Mass Entrepreneurship and Innovation” in 2014 in the hope to promote the smooth transition of China’s economy through innovation and entrepreneurship. Since enterprises usually have more internal resources and support, entrepreneurship within the enterprise can often create more valuable products and services under the support of favorable scenarios (Chen, 2019; Barraza et al., 2021). In the context of the new normal economy, enterprises should be in the vanguard of innovation and entrepreneurship, since their innovative and entrepreneurial behavior has vital practical significance for the development of the national economy (Clara et al., 2020; Deng et al., 2021).

Qian et al. (2018) found that empowered leadership was positively correlated with feedback seeking of followers, and employee feedback seeking was positively correlated with task performance, leader, and voice. In addition, employee feedback seeking mediated the positive relationship between empowered leadership and task performance, leader, and voice (Qian et al., 2018). Wu et al. (2019) took MBA students from Tianjin University as samples to analyze the nexus among the dark triad, entrepreneurial self-efficacy (ESE), and entrepreneurial intention (EI). They found that the dark triad positively predicted EI, and ESE had a partial mediating effect on the dark triad and EI. Moreover, narcissism/psychopathy had a negative effect on ESE and EI; narcissism/psychopathy had a non-linear effect on EI. Besides, Machiavellianism had a positive impact on ESE and EI. ESE had a mediating effect on the three members of the dark triad and EI (Wu and Song, 2019). Ding (2020) stated that the ability of learners to work together and coordinate efforts in a team was becoming increasingly important to the success of any work and progress in knowledge (Ding, 2020). Wu et al. (2020) found that the internal and external networks of business incubators positively affect enterprise growth performance, and exploratory learning and exploitative learning mediate the relationship between the two. Moreover, when incubators were in a highly dynamic environment, internal networks more positively affected exploitative learning, while external networks inhibited exploratory learning (Wu et al., 2020). Feng and Chen (2020) constructed a model of the relationship between entrepreneurial passion and psychology and behavior of entrepreneurs, put forward relevant hypotheses, and proposed a promotion mechanism based on the theory of self-efficacy (Feng and Chen, 2020). Douglas et al. (2021) discussed the problems of environmental cost control mode and the relationship between the value chain and environmental cost control. Besides, they applied the decision tree algorithm of artificial intelligence in designing the environmental cost control system of manufacturing enterprises to realize the internalization of environmental costs (Douglas et al., 2021). Deng et al. (2021) constructed a multi-level mediation model of the relationship between perceived environmental vitality and innovation of entrepreneurial team members based on the uncertainty reduction theory (Deng et al., 2021). A total of 235 articles are retrieved in China National Knowledge Infrastructure, Wanfang Database, and other databases. According to the inclusion and exclusion criteria here, 18 articles are finally selected in the introduction.

This study aims to explore the influencing mechanism of model innovation level on entrepreneurial behavior. First of all, the direct and indirect influences of model innovation on entrepreneurial behavior are investigated based on news communication theory. Meanwhile, ESE is taken as a mediator variable to explore how model innovation affects ESE to further study how ESE affects entrepreneurial behavior. Secondly, organizational commitment is utilized as a moderator to explain the moderating effect of organizational commitment to model innovation and entrepreneurial behavior and the moderating effect of organizational commitment to ESE and entrepreneurial behavior.

The entrepreneurial behavior and model innovation of new ventures are investigated based on news communication to reveal the importance of entrepreneurial behavior and model innovation for new ventures and provide the momentous management implications for entrepreneurial activities of enterprises. This investigation helps enterprises to understand the causes of entrepreneurial behavior and improve their efficiency and effectiveness. The innovation of this study is to break through the limitations of entrepreneurial behavior and clarify the connotation of entrepreneurial behavior and model innovation. The existing research focuses on the integrated implementation of entrepreneurial behavior, which is limited to the behavioral level, ignoring the behavioral logic, and unable to clarify the reasons for the phased display and transformation of entrepreneurial behavior.

The practical significance of this work is as follows. From the perspective of corporate performance, this work helps enterprises to understand the causes of entrepreneurial behavior within the company from another perspective, provides a new foundation for enterprises to attract, and retains creative talents. Besides, it offers new possibilities to enterprises to improve efficiency and effectiveness from the perspective of innovation and entrepreneurship. From the perspective of enterprise management, this study is conducive to understanding the ability of employees and provides a basis for the study of the impact of the ability of employees on internal entrepreneurship behavior. Besides, it provides a reference for strengthening the cultivation of creativity by enterprises based on the intermediary role of ESE and provides new possibilities for enterprises to implement entrepreneurship. From the perspective of employees, this study helps employees to understand the management behavior of enterprises and view the entrepreneurial behavior within enterprises from the perspective of employees, and it also provides the basis for employees to spontaneously carry out creative activities.

## Related Theories, Research Hypotheses, and Research Model

### Theories Concerning News Communication

News is a report on recent facts (Fisher et al., 2020). News is a linguistic statement of facts, so facts are primary, followed by reports (Francesco et al., 2020). No matter how media technology evolves, the essence of news remains to be reporting on recent facts (Hubner et al., 2021). The value elements of news are still critical criteria for people to measure the value of a news release, namely, the authenticity, timeliness, essentiality, distinction, proximity, and attractiveness of news (Miho, 2020).

Professor Shao Peiren of Zhejiang University defines communication as an activity in which people exchange information through symbols and media and expect some changes. Communication includes interpersonal communication, group communication, and mass communication (Wang, 2021). News is the public dissemination of information about recently varying facts (Rogoza et al., 2018; Sabah and Sarwar, 2021). This definition indicates that newly changed facts are the basis and sources of news and emphasizes the prerequisite and decisive role of facts in news communication activities. In essence, news communication is a reflection of the human subjective spirit of objective existence and a way for human cognitive activities to grasp the objective world (Schickinger et al., 2021; Stappers and Andries, 2021). Moreover, the mode of news communication is a simple way to understand and express the nature and basic laws of things (Su et al., 2021; Vladislav and Luo, 2021).

The fundamental social value of news communication is to protect the interests of the public to promote social development. From the perspective of the history of human development, the change of ideas is the forerunner of social change, but sometimes it cannot directly promote social progress. Ideas can be converted into materials only under the guidance of advanced ideas to form a good system and proper methods (Wu and Wu, 2017). The realization of the social value of news communication should be based on society, rather than confined to news communication itself. The most vital role of news communication is to reveal the latest factual information to the public and influence them (Pei et al., 2020).

### Theories Concerning Corporate Entrepreneurial Behavior

The theory of corporate entrepreneurship was first proposed in 1983, and since then a new research focus has been opened up for entrepreneurship research. Scholars have begun to study entrepreneurial activities from the perspective of the organization (Song et al., 2020). There are two types of corporate entrepreneurship: first, create new business within the existing organization; second, organizational change, also called the strategic renewal of organizations (Li et al., 2020). On the one hand, some scholars believe that corporate entrepreneurship is to create new organizations or promote the innovation of existing organizations according to the specific circumstances of existing organizations (Wang and Chen, 2020). On the other hand, it is believed that corporate entrepreneurship refers to entrepreneurial activities that create new businesses within the organization, open new businesses outside the organization, or establish new strategic alliances and even establish new joint ventures (Laviada et al., 2020; Li and Xu, 2020). Additionally, some scholars believe that corporate entrepreneurship is a strategic behavior from the perspective of strategic behavior, reflecting the characteristics of innovation in creating, utilizing, or expanding resources (Michael et al., 2020). The classification of entrepreneurial behavior is represented in Table 1.

**TABLE 1:** Classification of entrepreneurial behavior.

	Entrepreneurship
**Individual entrepreneurship**	
Corporate entrepreneurship	Intrapreneurship; strategic renewal

As shown in Table 1, entrepreneurship contains personal entrepreneurship and corporate entrepreneurship, and corporate entrepreneurship is further divided into internal entrepreneurship and strategic renewal of the organization. The behavior of strategic renewal may both occur outside the corporate and within the corporate. Therefore, the definition of corporate entrepreneurial behavior includes business innovation behavior within the organization and strategic updating outside the organization.

### Self-Efficacy Theory

Self-efficacy theory is an essential part of Bandura’s social cognitive theory. It is defined as the self-judgment of the expected results of a series of activities carried out by a specific organization in a particular situation. It emphasizes that different organizations will produce different self-efficacy in different situations, and self-efficacy has a contingency. Self-efficacy is also the self-cognition of the individual, who receives information from his/her own internal characteristics and produces self-efficacy through cognitive response. Therefore, self-efficacy is a specific manifestation of cognition. There are four influencing factors of self-efficacy, namely, achievement performance, alternative experience, speech persuasion, and physiological emotional state. The achievement performance means that personal experience and success or failure experience related to specific activities will have a great impact on self-efficacy. Alternative experience indicates that observing others for symbolic imitation will affect the formation of self-efficacy. Speech persuasion shows that persuasion through the facts of others will affect the formation of self-efficacy. The physiological emotional state also affects self-efficacy to some extent. Self-efficacy theory believes that self-efficacy has an impact on behavior choice, behavior performance, and resilience of people. Individuals with high self-efficacy will take positive measures to achieve the desired goal, while individuals with low self-efficacy tend to take avoidance measures.

### Research Hypotheses

According to relevant research literature, model innovation has a certain impact on the entrepreneurial behavior of new ventures based on the perspective of news communication. Besides, ESE as a mediator variable has a significant impact on entrepreneurial behavior. Meanwhile, organizational commitment is tentatively used as the moderator between model innovation and entrepreneurial behavior, and the moderator between ESE and entrepreneurial behavior. Moreover, the organizational commitment is divided into three dimensions to deeply explore the moderating effects of continuance commitment, normative commitment, and affective commitment under organizational commitment through the study of different dimensions. Therefore, the following assumptions can be summarized.

Hypothesis 1: Model innovation has a positive impact on entrepreneurial behavior.
Hypothesis 2: Model innovation has a positive impact on ESE.
Hypothesis 3: Organizational commitment positively regulates the relationship between model innovation and entrepreneurial behavior. The higher the level of organizational commitment, the greater the impact of model innovation on entrepreneurial behavior.
Hypothesis 4: Normative commitment positively regulates the relationship between model innovation and entrepreneurial behavior. The higher the level of normative commitment, the greater the impact of model innovation on entrepreneurial behavior.

### Research Model

**FIGURE 1:**
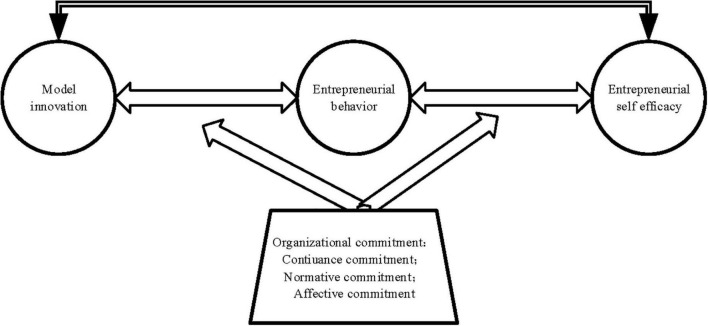
Theoretical research model.

Figure 1 illustrates the research model reported here.

According to Table 2 and Figure 1, Hypothesis 1 is set as the impact of model innovation on entrepreneurial behavior, and Hypotheses 2 and 3 concern the mediating effect of ESE. Hypothesis 4 is related to the moderating effect of organizational commitment. The model innovation described in Figure 1 has a significant positive effect on entrepreneurial behavior and ESE. Combined with the hypotheses in Table 2, evidently, the higher the level of organizational commitment, the greater the impact of model innovation on entrepreneurial behavior. Besides, the higher the level of normative commitment, the greater the impact of model innovation on entrepreneurial behavior.

**TABLE 2:** Questionnaire.

No.	Question	Totally disagree	Disagree	Basically agree	Agree	Totally agree
1	I can identify the potential value of an idea.					
2	I can present good ideas for new products or services to senior management in new market environment.					
3	I can motivate members of my department or team with new business ideas					
4	I am often proactive in communicating with others to accurately estimate the cost of new projects being run.					
5	I have creative ideas in my work and put them into action.					
6	I take the initiative to explore new technologies and products.					
7	I can identify long-term weaknesses and future opportunities.					
8	I can create technology or value-added services for new products to make them more competitive.					
9	I can obtain cooperation opportunities with competitive prices and contracts					
10	I can make a clear plan for the future development of the department or team.					

### Data Collection by the Questionnaire

A total of 150 questionnaires are collected through an online medium, such as WeChat and e-mail. There are a total of 136 valid questionnaires after excluding the questionnaires under excessively short answer time or with extreme scores, with an efficiency of 90.67%. The data from 136 valid questionnaires are used for further analysis.

### Questionnaire Test

Reliability refers to the stability, dependability, and consistency of the questionnaire measurement results. The higher the reliability of the measurement, the more credible the measurement results. Table 3 reveals the results of the reliability test by inputting the sample data into SPSS25.0 for analysis.

**TABLE 3:** Reliability test results.

Variable	Cronbach α
Entrepreneurial behavior	0.904
Entrepreneurial self-efficacy	0.947
Model innovation	0.913
Continuance commitment	0.887
Normative commitment	0.883
Affective commitment	0.852

In Table 4, the Cronbach α coefficient of model innovation, ESE, and entrepreneurial behavior is >0.9, which belongs to excellent reliability. The Cronbach α coefficient of continuance commitment, normative commitment, and affective commitment is >0.8, indicating that the reliability is excessively brilliant. In summary, the reliability of the questionnaire fully meets the measurement standard.

**TABLE 4:** Test results of questionnaire reliability.

Variable	Cronbach α
Model innovation	0.903
Entrepreneurial self-efficacy	0.947
Entrepreneurial behavior	0.911
Continuance commitment	0.887
Normative commitment	0.868
Affective commitment	0.841

### Regression Analysis of the Moderating Effect of Organizational Commitment

The organizational commitment is divided into three dimensions here, namely, continuance commitment, normative commitment, and affective commitment, intending to verify the moderating effect of organizational commitment on entrepreneurial behavior and model innovation according to these three dimensions. Before analyzing the moderating effect, it is necessary to centralize model innovation, ESE, continuance commitment, emotional commitment, and normative commitment.


(1) Z⁢X⁢W⁢1=Contiuance⁢commitment×Model⁢innovation

(2) Z⁢M⁢W⁢1=C⁢ontinuance⁢commitment×Entrepreneurial⁢self-efficacy

(3) Z⁢X⁢W⁢2=Normative⁢commitment×Model⁢innovation

(4) Z⁢M⁢W⁢2=Normative⁢commitment×Entrepreneurial⁢self-efficacy

The principle of centralization is to subtract the mean value of each variable and multiply the interaction terms of the five centralized variables to obtain the centralized product.

## Data Analysis and Discussion

### Descriptive Statistical Analysis of Survey Subjects

**FIGURE 2:**
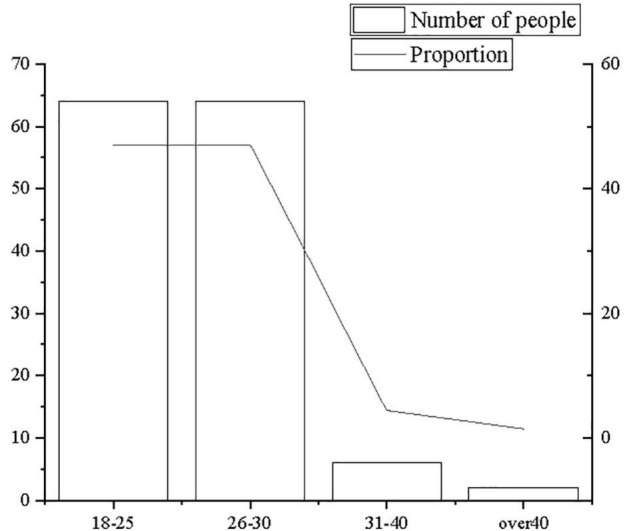
Statistics of age distribution.

**FIGURE 3:**
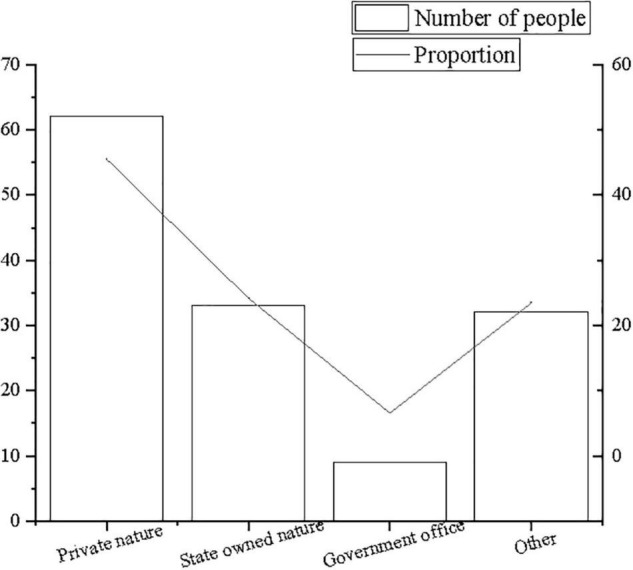
Statistics of distribution of company nature.

**FIGURE 4:**
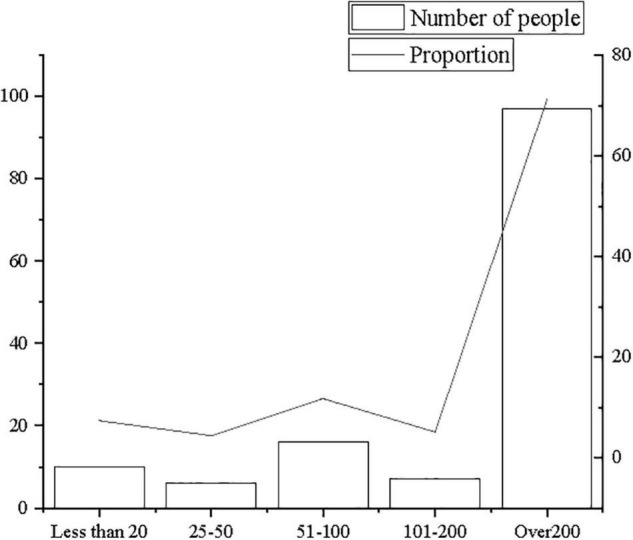
Statistics of distribution of company size.

The descriptive statistics of survey subjects principally focus on the age of survey subjects, the nature of the company, and the company size to carry out quantitative analysis and percentage analysis, as shown in Figures 2–4.

From Figures 2–4, the majority of research subjects are 18–25 years old or 26–31 years old, which is consistent with the research focus. Most employees aged 18–31 years old are in the initial stage of their careers, and they are mostly in the rank-and-file positions of the company. In terms of company nature and company size, the majority are private companies and state-owned companies, which are large- and medium-sized companies with more than 200 people, in line with the basic situation of corporate entrepreneurship. Therefore, it is believed that the sample structure is comparatively reasonable.

### Descriptive Statistical Analysis of Variables

**FIGURE 5:**
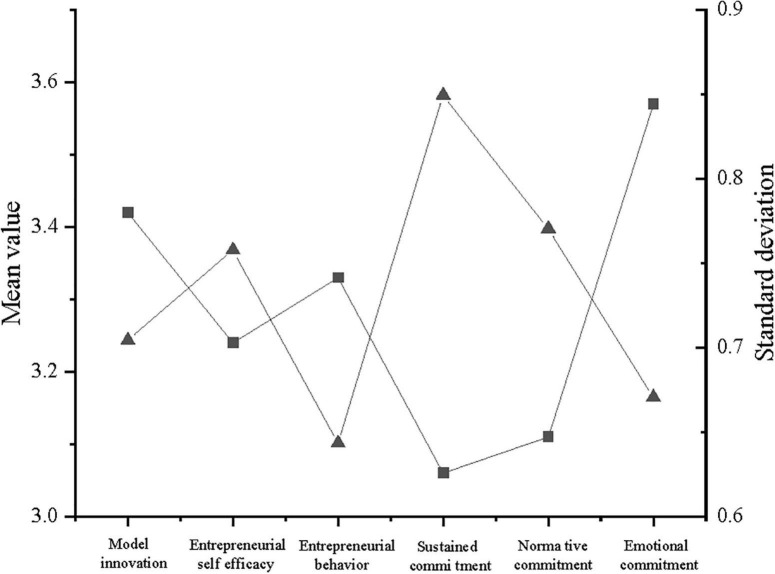
Descriptive statistics of variables.

Descriptive statistics of variables describe the maximum value, mean value, and SD of variables, to understand the variables and related basic characteristics. Figure 5 illustrates the descriptive statistical mean value and SD of model innovation, ESE, entrepreneurial behavior, continuance commitment, normative commitment, and affective commitment.

According to Figure 5, the mean value of model innovation is 3.42, which is above the median of the data, indicating that the samples have a high average level of model innovation. Similarly, the mean value of ESE is 3.24, which is above the median of the data, suggesting that the samples have a high level of ESE. Moreover, the mean value of entrepreneurial behavior is 3.33, which is also higher than the median of the data. The descriptive description shows that the level of entrepreneurial behavior in the sample is high. Besides, the mean value of continuance commitment is 3.06, which is higher than the median of the data, signifying that the samples have a high level of continuance commitment. Furthermore, the mean value of normative commitment is 3.11, which is above the median of the data. The descriptive description shows that the level of normative commitment in the sample is high. Finally, the mean value of affective commitment is 3.57, which is above the median of the data, showing that the level of affective commitment in the sample is high.

### Reliability Test

Reliability refers to the stability and consistency of the measurement results. The higher the reliability of the measurement is, the more credible the measurement results are. Table 3 demonstrates the analysis results of the sample data by SPSS25.0 software.

In Table 3, the Cronbach α values of model innovation, ESE, and entrepreneurial behavior are >0.9, showing excellent reliability. The Cronbach α values of continuance commitment, normative commitment, and affective commitment are >0.8, indicating that the questionnaire has excellent reliability. In summary, the reliability of the questionnaire fully meets the measurement standard.

### Validity Test

Validity denotes the availability of measurement. Since domestic and foreign scales that are relatively mature are adopted in the questionnaire survey, confirmatory factor analysis (CFA) is adopted to verify the structural validity of the research model. Table 5 illustrates elementary data obtained through the analysis of AMOS24.0.

**TABLE 5:** Statistics data of all variables and related dimensions.

Fitting index	Value
Overall fitting index	2.01
RMSEA	0.06
CFI	0.89
TLI	0.78

The CFA results of six variables, namely, six factors, reveal that several key data are relatively more than or close to the standard value. The overall fitting index of the research model is 2.021, close to the standard value 2; the root-mean-square error of approximation (RMSEA) value is 0.059, slightly higher than the standard value of 0.05; the comparative fit index (CFI) value is 0.903, slightly greater than the standard value of 0.9; and the Tucker-Lewis index (TLI) value is 0.896, slightly lower than the standard value of 0.9. These results indicate that the constructed model is within an acceptable range. In other words, the validity of adopted scales meets the research standard, and there is a good distinction between the variables.

**FIGURE 6:**
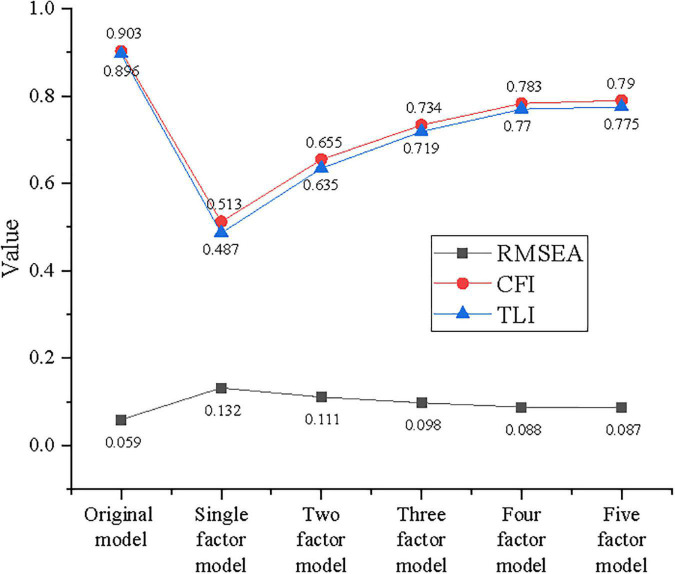
Confirmatory factor analysis (CFA) of the competition model.

Figure 6 displays the test results of the discriminant validity comparing the competition model with the original model.

Figure 6 illustrates that with comparative analysis of the competition model, RMSEA values of all models are >0.05, and all CFI and TLI values are <0.9. This indicates that there are indeed differences between model innovation, ESE, entrepreneurial behavior, continuance commitment, normative commitment, and affective commitment. Besides, various dimensions of variables show the acceptable distinction. This lays the groundwork for subsequent correlation analysis and regression analysis and also verifying the effectiveness of subsequent research.

### Correlation Analysis

**FIGURE 7:**
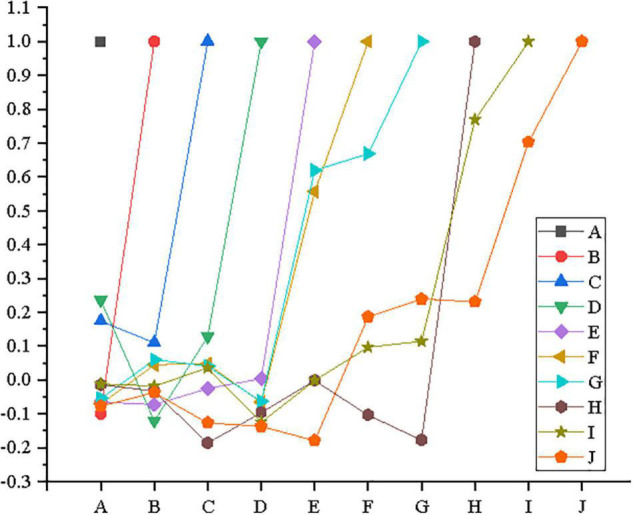
Correlation analysis among variables (A: educational background; B: employees’ gender; C: employees’ age; D: company size; E: model innovation; F: entrepreneurial self-efficacy; G: entrepreneurial behavior; H: continuance commitment; I: normative commitment; and J: affective commitment).

Based on the reliability and validity analysis of the formal questionnaire, the statistical methods of correlation analysis and regression analysis are employed to test the research hypotheses reported above. The correlation analysis results of variables by IBM SPSS25.0 are shown in Figure 7.

In Figure 7, the correlation coefficient between any variable and itself is 1. Among the control variables, age and continuance commitment of employees are significantly correlated above 0.01 with a correlation coefficient of −0.186. Company size and continuance commitment, normative commitment, and affective commitment are significantly correlated above 0.05 with the correlation coefficients of −0.096, −0.124, and −0.137, respectively.

In addition to control variables, the correlation coefficient between model innovation and ESE is 0.557, which was significantly correlated above 0.01. The correlation coefficient between model innovation and entrepreneurial behavior is 0.620, which is significantly correlated above 0.01. The correlation coefficient between ESE and entrepreneurial behavior is 0.669, significantly correlated above 0.01. The correlation coefficients between model innovation and continuance commitment, normative commitment, and affective commitment are −0.001, −0.001, and −0.179, respectively, and the correlation coefficients between model innovation and affective commitment are significantly correlated above 0.01. The correlation coefficients between ESE and continuance commitment, normative commitment, and affective commitment are −0.103, 0.097, and 0.187, respectively, and ESE and emotional commitment are significantly correlated above 0.01. The correlation coefficients between entrepreneurial behavior and continuance commitment, normative commitment, and affective commitment are −0.177, 0.114, and 0.239, respectively. Meanwhile, entrepreneurial behavior and continuance commitment or affective commitment are significantly correlated above 0.01.

There is also a correlation between continuance commitment and normative commitment or between continuance commitment and affective commitment. The correlation coefficients between continuance commitment and normative commitment and between continuance commitment and affective commitment are 0.077 and 0.231, respectively. The correlation between continuance commitment and affective commitment is significantly correlated above 0.01. The correlation coefficient between normative commitment and affective commitment is 0.703, and it is significant above 0.01.

In summary, the analysis results are highly consistent with the research hypotheses above.

### Regression Analysis of the Direct Effect of Model Innovation on Entrepreneurial Behavior

**FIGURE 8:**
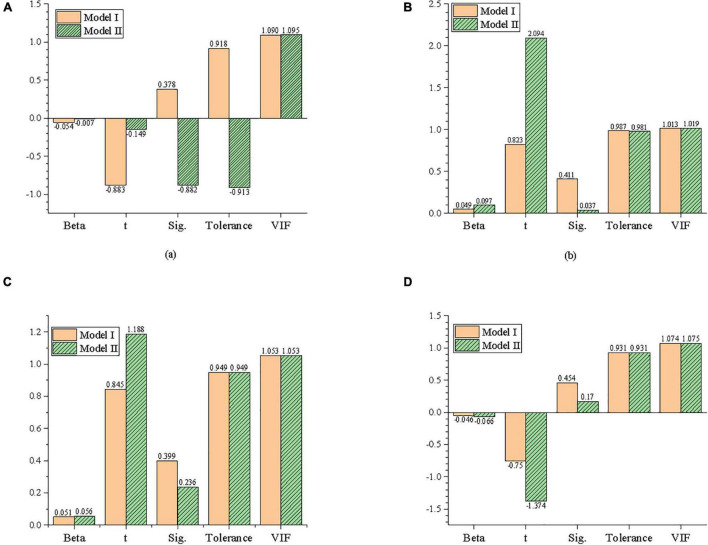
Regression analysis of the effect of model innovation on entrepreneurial behavior. **(A)** Educational background, **(B)** gender, **(C)** age, and **(D)** company size.

Firstly, the statistical variables, such as educational background and gender in model innovation, are taken as independent variables, and entrepreneurial behavior is taken as the dependent variable to establish Model I. Secondly, the educational background, gender, and other statistical variables are taken as control variables, model innovation is taken as independent variables, and entrepreneurial behavior is taken as dependent variables to establish model II. The results of regression analysis through IBM SPSS25.0 are exhibited in Figure 8.

Figure 8 reveals that the statistical variables in Model I all show light influences. When the model innovation is put into Model II, gender has a significant impact on entrepreneurial behavior. Adjusting R^2^ increases from −0.04 to 0.389, indicating that model innovation can explain 38.9% of the variance of entrepreneurial behavior, and the proportion of explained variance grows significantly. Meanwhile, the value of the F test is 37.736, which is statistically significant and significantly improved. This indicates that the regression equation is effective, and Model II has a better fitting efficiency than Model I. The standardization regression coefficient Beta of model innovation is 0.628, and the *t* value of significance test is 13.557. This indicates that model innovation has a significant positive impact on entrepreneurial behavior, which supports Hypothesis 1.

Moreover, the Variance Inflation Factor (VIF) values of control variables and independent variables are between 1 and 2, indicating that there is no collinearity between independent variables and control variables, i.e., educational background, gender, age, and company size.

### Regression Analysis of the Effect of Model Innovation on Entrepreneurial Self-Efficacy

**FIGURE 9:**
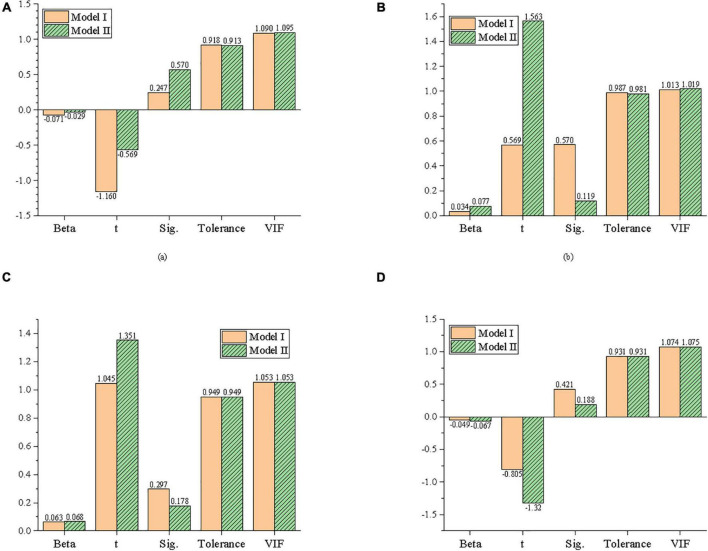
Regression analysis of the effect of model innovation on entrepreneurial self-efficacy. **(A)** Educational background, **(B)** gender, **(C)** age, and **(D)** company size.

Firstly, the education level, gender, age, and company size of statistical variables in model innovation are taken as independent variables, with ESE as dependent variables to establish model I. Secondly, the educational background, gender, and other statistical variables are taken as control variables, with model innovation as independent variables and ESE as dependent variables to establish model II. Figure 9 represents the regression analysis results by IBM SPSS25.0.

From Figure 9, the influence of statistical variables in Model I is not significant. Meanwhile, the influence of each statistical variable is not significant after putting model innovation into Model II. Adjusting R^2^ increases from −0.01 to 0.313 indicating that model innovation can explain 31.3% of the variance of ESE and the proportion of explained variance increases significantly. Besides, the value of the F test is 27.232, which is statistically significant and obviously improves, indicating that the regression equation is effective, and the fitting efficiency of Model II is better than that of Model I. The standardization regression coefficient Beta of model innovation is 0.562, and the *t*-value of the significance test is 11.441. This shows that model innovation has a significant positive impact on ESE, which is a coefficient with Hypothesis 2.

Furthermore, the VIF values of both control variables and independent variables are between 1 and 2, indicating that there is no collinearity between the independent variable and the control variables, such as educational background, gender, age, and company size.

### Regression Analysis of the Moderating Effect of Continuance Commitment on Model Innovation and Entrepreneurial Behavior

**FIGURE 10:**
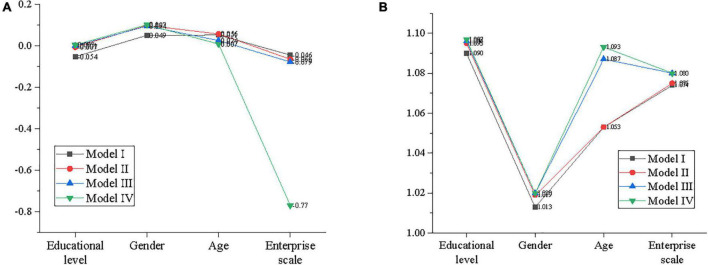
Regression analysis of the moderating effect of continuance commitment on model innovation and entrepreneurial behavior. **(A)** Beta value, **(B)** variance inflation factor (VIF) value.

Educational background and gender in model innovation are selected as control variables for the regression analysis by IBM SPSS25.0. First, the educational background, gender, and other statistical variables are seen as independent variables with entrepreneurial behavior as the dependent variables to establish model I. Then, the educational background, gender, and other statistical variables are regarded as control variables, with model innovation as independent variables and entrepreneurial behavior as dependent variables, to establish model II. Then, educational background, gender, and other statistical variables are taken as control variables, with model innovation as independent variables, while continuance commitment as independent variables, to construct model III. Finally, statistical variables, such as educational background and gender, are taken as control variables, with model innovation as independent variables and continuance commitment as independent variables. The decentralized cross-product term (ZXW1) of continuance commitment and model innovation are taken as independent variables, with entrepreneurial behavior as dependent variable, to build model IV. Figure 10 reveals the overall analysis results.

According to Figure 10, the adjustment R^2^ increases from 0.418 in Model III to 0.467 in Model IV, indicating that the ability of the interaction term to explain the variance of entrepreneurial behavior is improved after being put into Eq. (1). Meanwhile, the F test is also statistically significant, indicating that model IV has a better fitting degree than model III. Besides, the interaction term between model innovation and continuance commitment in the equation has a significant explanatory capability for the entrepreneurial behavior of the employee. Additionally, the regression coefficient of the interaction term between model innovation and continuance commitment (ZXW1) is statistically significant, indicating that continuance commitment plays a regulatory role in the process of model innovation affecting the entrepreneurial behavior of the employee, which verifies Hypothesis 3.

Furthermore, the VIF values of explanatory variables are between 1 and 2, indicating that there is no collinearity between explanatory variables.

### Regression Analysis of the Moderating Effect of Normative Commitment on Model Innovation and Entrepreneurial Behavior

**FIGURE 11:**
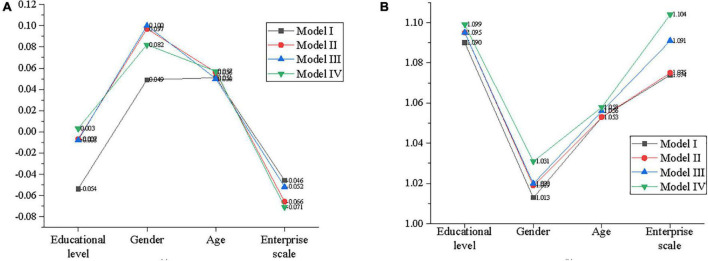
Regression analysis of the moderating effect of normative commitment on model innovation and entrepreneurial behavior. **(A)** Beta value, **(B)** variance inflation factor (VIF) value.

First of all, the educational background, gender, age, company size, and other statistical variables are regarded as independent variables, with entrepreneurial behavior as dependent variables, to establish model I. Then, the educational background, gender, and other statistical variables are taken as control variables, with model innovation are taken as independent variables and entrepreneurial behavior as dependent variables, to establish model II. Next, the educational background, gender, and other statistical variables are seen as control variables, with model innovation is seen as independent variables and normative commitment is as independent variables, to build model III. Finally, statistical variables, such as educational background and gender, are taken as control variables, with model innovation is taken as independent variables and normative commitment is taken as independent variables. The decentralized cross-product term (ZXW2) of normative commitment and model innovation is taken as the independent variables, and entrepreneurial behavior is taken as the dependent variable, to build model IV. Figure 11 signifies the regression analysis results by IBM SPSS25.0.

According to Figure 11, the adjustment R^2^ increases from 0.399 in Model III to 0.428 in Model IV, indicating that the ability of interaction term in Eq. (4) to explain the variance of entrepreneurial behavior is improved after being put into the equation. Meanwhile, the F test is also statistically significant, which shows that model IV has a better fitting degree than model III. Besides, the interaction term of model innovation and normative commitment (ZXW2) put into the equation has a significant explanatory capability for the entrepreneurial behavior of the employee. Additionally, the regression coefficient of the interaction term between model innovation and normative commitment (ZXW2) is significant. This reveals that normative commitment plays a regulatory role in the process of model innovation affecting employee entrepreneurial behavior, which supports Hypothesis 4.

Moreover, the VIF values of explanatory variables are between 1 and 2 (VIF < 5), indicating that there is no collinearity between explanatory variables.

## Conclusion

Based on news communication theory and social cognitive theory, a theoretical model is constructed here to analyze the interaction effects of entrepreneurial behavior, ESE, and organizational commitment. First, the influence mechanisms of ESE and organizational commitment between model innovation and entrepreneurial behavior are expounded. Then, a series of hypothetical propositions are put forward. Finally, an empirical analysis is performed on the theoretical model by effective sample data, and the main conclusions are as follows: (1) model innovation can significantly promote entrepreneurial behavior; (2) ESE plays an intermediary role between model innovation and entrepreneurial behavior; (3) continuance commitment moderates the relationship between model innovation and entrepreneurial behavior; and (4) normative commitment regulates the relationship between model innovation and entrepreneurial behavior.

However, due to the limitation of article length and research time, there are still some deficiencies to be improved in this work. On the one hand, the experimental samples are primarily collected from the own social network of the author, and the samples are not selected randomly, which may lead to incomplete data. In the future, the source of samples will be expanded to ensure the diversification of research data and make the research conclusions more universal. On the other hand, there are only 136 valid questionnaires, and the number of entrepreneurs and company managers is insufficient. The follow-up research needs to further explore the control variables.

Through questionnaire survey and data analysis, it can conclude that model innovation and ESE can significantly promote entrepreneurial behavior. This work enriches the quantitative research results of the antecedents of entrepreneurial behavior, expands the research content of corporate entrepreneurship, and reveals the significance of entrepreneurial ability, ESE, and organizational commitment to the implementation of entrepreneurial behavior. Besides, it provides a critical reference for the implementation of entrepreneurial behavior and the improvement of the performance of start-ups and also offers vital management enlightenment for the entrepreneurial activities of enterprises. Future research can deeply investigate the impact of ESE and organizational commitment on corporate entrepreneurial behavior and model from a broader perspective and multiple regulatory variables to achieve more diversified research results.

## Data Availability Statement

The raw data supporting the conclusions of this article will be made available by the authors, without undue reservation.

## Ethics Statement

The studies involving human participants were reviewed and approved by the Southwestern University of Finance and Economics Ethics Committee. The patients/participants provided their written informed consent to participate in this study. Written informed consent was obtained from the individual(s) for the publication of any potentially identifiable images or data included in this article.

## Author Contributions

All authors listed have made a substantial, direct and intellectual contribution to the work, and approved it for publication.

## Conflict of Interest

The authors declare that the research was conducted in the absence of any commercial or financial relationships that could be construed as a potential conflict of interest.

## Publisher’s Note

All claims expressed in this article are solely those of the authors and do not necessarily represent those of their affiliated organizations, or those of the publisher, the editors and the reviewers. Any product that may be evaluated in this article, or claim that may be made by its manufacturer, is not guaranteed or endorsed by the publisher.
